# Assessment of Knowledge, Attitude, Practise and Health Literacy (KAPH) Towards COVID-19 in Post-COVID-19 New Reality: The Need and Its Challenges in Malaysia

**DOI:** 10.3389/fpubh.2021.704115

**Published:** 2021-07-23

**Authors:** Izzaty Dalawi, Xin Wee Chen, Mohamad Rodi Isa, Zahir Izuan Azhar, Fadzilah Mohd Nor

**Affiliations:** ^1^Department of Public Health Medicine, Faculty of Medicine, Universiti Teknologi MARA, Sungai Buloh Campus, Sungai Buloh, Malaysia; ^2^Ministry of Health, Putrajaya, Malaysia; ^3^Department of Medical Microbiology and Parasitology, Faculty of Medicine, Universiti Teknologi MARA, Sungai Buloh Campus, Sungai Buloh, Malaysia; ^4^Integrative Pharmacogenomics Institute (iPROMISE), Universiti Teknologi MARA, Puncak Alam Campus, Selangor, Malaysia

**Keywords:** knowledge, health literacy, practise, COVID-19, pandemic (COVID-19), Malaysia

## Introduction

Coronavirus disease 2019 (COVID-19) is a public health emerging infection globally with a terrifying impact. The rise in COVID-19 cases is still alarming, despite many preventive and control measures implemented, including the recent “promotion” of herd immunity. This emergence caused by a newly discovered coronavirus has alerted the Malaysian health system on preparedness to combat a pandemic crisis. Despite the acceleration of vaccine development, Malaysians must adjust to a new normal post-COVID-19, as, thus far, it is still the best preventive measure at the population level. Many countries, including Malaysia, succeeded in flattening the curve for the earlier waves and achieved zero new cases at one point in time; however, the latest deterioration of the transmission of the disease, the rise in COVID-19 cases and the death toll have put a great concern to all countries, including Malaysia (1).

This study aims to describe the indications for the need to assess knowledge, attitude, practise and health literacy (KAPH) of Malaysians towards COVID-19 including (i) analysing factors that attribute to the trend of KAPH level, (ii) providing the baseline for the effect of vaccination and (iii) imparting crucial tool to be incorporated into the new home quarantine policy in Malaysia and the anticipated challenges for such assessment in the post-COVID-19 era. The topic is critically important but is often neglected in the decision-making process to support the management of COVID-19 and vaccination strategies.

To date, there were three substantial indicators observed by the authors including the following:

### Public Compliance Towards the COVID-19-Related Standard Operating Procedures (SOPS)

The KAPH levels affect the public compliance towards the COVID-19-related SOPs. This pandemic has gotten worse as compared to when it was first started, perhaps due to the suboptimal awareness of the disease within the community. The sudden surge of daily cases since October 2020 triggered the Malaysian government to announce the implementation of Movement Control Order (MCO) 2.0 from 13 January 2021, which is a more targeted version of the MCO implemented in March 2020, considering the impact on livelihoods of people and the economic stability of the country. Even though the current daily confirmed cases showed a reduction in trend starting the end of February 2021, we could visualise the recent increasing trend of reproduction number that is worrisome (2). With many sectors opening recently, including the schools and stores, this may pose the rise of risk of transmission in the community. Thus, public compliance towards the SOPs is substantial in affecting the changes in the infectivity trend. We need to assess the KAPH of the community on COVID-19 from time to time and analyse factors related to the trend of KAPH level, as these may assist public health authorities to plan and implement the appropriate preventive and control measures accordingly. Besides that, fake news remains a significant concern to the public health fraternity as this could lead to grave misunderstanding and confusion. Subsequently, this will affect their attitudes and how people react to COVID-19. This issue also could seriously affect the level of KAPH among the public if it is not handled effectively.

### Measurement of Vaccination Effect

The assessment of KAPH is suggested before an intervention/vaccination programme. The assessment will mark the baseline prior to measurement of the vaccination effect soon after its introduction, especially in the third phase of the National COVID-19 Immunisation Programme of Malaysia, which tentatively will be given starting from May 2021 to February 2022 (3, 4). In Malaysia, vaccines are delivered in three phases. Phase 1 focused on the frontliners including healthcare personnel and those involved in essential services such as defence and security forces. Phase 2 prioritises the remainder of healthcare workers, senior citizens aged 60 years and above, high-risk groups with chronic diseases and people with disabilities. Phase 3 will cover the vast remainder of the public aged 18 years and above regardless of their citizenship (4). At the time of writing, Malaysia has only hit the first phase and it is still early to announce that the reduction in the number of confirmed daily COVID-19 cases attributed to the commencement of vaccine policy in Malaysia. The emergence of anti-vaccination or attitude of vaccine reluctance within the general population should be considered as a threat as these may contribute to the diverse pattern of KAPH on COVID-19 in our community (5). Therefore, it is relevant to monitor the assessment of KAPH towards the COVID-19 prevaccination and postvaccination programme in our public. This is to ensure that the awareness regarding the importance of the behaviours of preventive measures is adapted into the new social norm.

The evolution of the COVID-19 pandemic is contributed by factors that will make any prediction of when the pandemic will end is scientifically undefinable (6). Despite the recent implementation of the vaccination programme, some have predicted the COVID-19 might end in the second half of 2021, and others predicted that the pandemic would remain at least up to the year 2022 or even later, depending on the efficacy, distribution and coverage of the vaccine (7, 8). In addition, the percentage of herd immunity to be achieved against the COVID-19 vaccine is still yet to be known and in doubt to achieve a good coverage taking into consideration those migrant groups, anti-vaccine / vaccine reluctance groups and population aged <18 years. Basic public health measures remained the most crucial entity; thus, the need for the assessment of KAPH among our community is highly needed from time to time.

### Home Quarantine Policy

The assessment of KAPH may become an essential tool to be incorporated into the new home quarantine policy in Malaysia. Malaysia has currently entered the mitigation phase of combating COVID-19 infection. Thus, on 13 January 2021, the Ministry of Health has instructed the home quarantine policy for close contacts of appropriate COVID-19-positive cases and only for those who are tested symptomatic (9). Furthermore, the positivity rate of Malaysia for the COVID-19 test has been reported to be more than 5% since November 2020 as recommended by the WHO (10). This reflects the undetected positive COVID-19 cases in the community, thus becoming unreported cases in Malaysia. The lesser the testing, the lesser confirmed cases to be detected and reported, hence the inaccurate rate of infectivity in the community. Consequently, this may produce misleading information to the community and policymakers since the wrong perception of the effectiveness of MCO 2.0 with the well-practised and compliance to the SOP begins to be taken lightly. It is only a matter of time before another wave of massive transmission of COVID-19 hits the country if the public becomes complacent. The effectiveness of the home quarantine policy depends mainly on self-compliant, self-awareness, KAPH on COVID-19 and its preventive measures. Good KAPH will lead to the success of the policy, reducing the transmission of COVID-19 and the burden to the healthcare system. Thus, assessment of KAPH is valid to be performed regardless of the vaccination programme that has already started, as this is to ensure the effectiveness of home quarantine policy and targeted educational programme to support KAPH that can be continuously improved from time to time.

### Challenges in the Assessment of KAPH

The authors anticipated two main challenges in assessing KAPH in Malaysia. The foremost hurdle would be to obtain a valid measurement tool of KAPH. In response to manage the COVID-19 pandemic effectively, the Malaysian government implemented several legislative interventions, including the imposition of different MCO levels, active case detection and provision of educational resources concerning COVID-19. Although numerous studies were conducted across the globe (11–14), and few conducted locally (15), to provide information on KAP to reveal the knowledge gaps and to advise the relevant authorities for targeted strategies, the findings of their study might not be applicable to the local setting, given the (i) non-pharmacological interventions implemented differently, compared to other countries (16), and (ii) the scarcity of reliable tool developed and validated in Malay (national language in Malaysia). Information on communication routes affected the awareness and knowledge of COVID-19 (14); hence, the health literacy component is recommended to be evaluated along with the KAP assessment.

The next obstacle would be to handle the possible selection bias in data collection. Due to the current situation, researchers tend to conduct online data collection; hence, volunteerism in answering the question, low response rate (among elderlies or technology-illiterate population or COVID-19 sceptic/denier) and the ability to include citizens from different socio-economic background might affect the representativeness of the data. A hybrid approach (face-to-face and online data collection) could minimise this systematic error.

## Conclusion

The empowerment of the community and continuous education programmes towards COVID-19 may serve as appropriate recommendations to educate the public regarding the importance of good compliance to the COVID-19-related SOPs. Thus, the need for an assessment of KAPH on COVID-19 in our community remains crucial and valid in the post-COVID-19 era. The assessment not only provides an insight into the understanding of the community of the disease but also continues to address the gap in improving KAPH and to ensure the preventive control measures that are effective in moderating the viral transmission. Furthermore, incorporating health literacy as part of the assessment is needed, for a better strategy emphasising the promotion of educational programmes related to COVID-19, and information sharing can be achieved and employed accordingly. Simultaneously, these may indirectly reduce the transmission of other communicable diseases. A standardised tool in assessing KAPH will enable the comparison to be made on KAPH between different settings and groups of people.

## Author Contributions

All authors contributed to the conceptualisation and writing of this manuscript.

## Conflict of Interest

The authors declare that the research was conducted in the absence of any commercial or financial relationships that could be construed as a potential conflict of interest.

## Publisher's Note

All claims expressed in this article are solely those of the authors and do not necessarily represent those of their affiliated organizations, or those of the publisher, the editors and the reviewers. Any product that may be evaluated in this article, or claim that may be made by its manufacturer, is not guaranteed or endorsed by the publisher.
